# Investigation of Adverse Events Associated with Predicted GnRHR Agonists Using the FAERS Database

**DOI:** 10.3390/ijms27156675

**Published:** 2026-07-27

**Authors:** Yui Migura, Yoshihiro Uesawa

**Affiliations:** 1 Department of Medical Molecular Informatics, Meiji Pharmaceutical University, Tokyo 204-8588, Japan; 2 Tokushima Research Center for Drug Discovery, Otsuka Pharmaceutical Co., Ltd., Tokushima 771-0192, Japan

**Keywords:** androgen deprivation therapy, anatomical therapeutic chemical, principal component analysis, primary suspect, preferred terms

## Abstract

Gonadotropin-releasing hormone receptor (GnRHR) agonists are widely used therapeutically, yet their adverse event profile remains insufficiently characterized. We developed a machine-learning model using Tox21 GnRHR agonist activity data and molecular descriptors to predict GnRHR agonist activity among FDA Adverse-Event Reporting System (FAERS)-listed drugs. BalancedRandomForest achieved the highest ROC-AUC (0.810) and was applied to 5523 FAERS-listed drugs. Following applicability-domain assessment, 1191 drugs were retained, of which 367 were predicted to have GnRHR agonist activity. FAERS data from 2004 Q1 through 2024 Q3 were analyzed using reporting odds ratios (RORs) and Fisher’s exact test for MedDRA Preferred Terms (PTs). Overall, 330 unique PTs corresponding to 531 PT–SOC assignments met prespecified criteria: at least 1000 reports, ln(ROR) > 1, and *q* < 0.05. The highest proportions of significant PTs were observed in respiratory, thoracic and mediastinal disorders; infections and infestations; and hepatobiliary disorders. These findings suggest that drugs predicted to have GnRHR agonist activity may show disproportionate reporting of respiratory, infectious, and hepatobiliary adverse events, although causality cannot be established. This framework may support hypothesis generation and safety signal prioritization during drug development and postmarketing surveillance.

## 1. Introduction

Gonadotropin-releasing hormone (GnRH) helps regulate the hypothalamic–pituitary–gonadal axis, which governs reproduction and sexual development. GnRH, produced by neurosecretory cells in the hypothalamus, binds to the GnRH receptor (GnRHR) expressed on gonadotroph cells in the anterior pituitary gland and acts in a pulsatile manner [1]. Consequently, the anterior pituitary gland secretes gonadotropins such as follicle-stimulating hormone and luteinizing hormone, which both act on the ovaries or testes and promote estrogen and testosterone production [2]. GnRH secretion is suppressed during childhood, but it increases at puberty to induce sexual development through increased gonadotropin and gonadal steroid production [3]. GnRHR signaling dysregulation can cause clinically important reproductive disorders, including polycystic ovary syndrome [4] and ovulatory dysfunction [5].

GnRHR agonists are therapeutic agents that are widely used for inducing ovulation in infertility treatment [6] and for treating prostate cancer [7], breast cancer [8], and endometriosis [9]. However, these drugs have been associated with adverse effects, including amenorrhea, hot flashes, and reduced bone mineral density [10]. More recently, they have been reported to be potentially associated with more serious adverse events, including interstitial lung disease [11] and liver disease [12]. Furthermore, a large-scale analysis using the Food and Drug Administration (FDA) Adverse-Event Reporting System (FAERS) suggested associations with reproductive system and breast disorders [13]. Nevertheless, previous studies mainly focused on specific drugs, and the overall adverse-event profile associated with GnRHR agonist activity remains insufficiently characterized.

The Toxicology in the 21st Century (Tox21) program was launched through a collaboration between the U.S. Environmental Protection Agency, the U.S. National Institutes of Health, and the U.S. FDA to promote the transition from conventional animal-dependent toxicity testing to in vitro assessment [14]. As part of this program, Yang et al. conducted comprehensive quantitative high-throughput screening of the Tox21 compound library against human and mouse GnRHRs in 2024; compounds with GnRHR agonist activity were identified from a broad chemical space comprising not only approved drugs but also environmental chemicals [15].

Large-scale in vitro datasets such as Tox21 provide an important foundation for constructing quantitative structure–activity relationship (QSAR) models. QSAR models predict biological activity from molecular structure, with recent studies integrating QSAR-based toxicity prediction with FAERS data [16]. However, the validity of the evaluation design must be given careful attention when assessing the QSAR model performance. Calle et al. reported that nested cross-validation reduces the performance estimate variance compared with single-split evaluation and avoids test-information leakage by tuning hyperparameters within an inner loop [17].

In this study, we aimed to clarify the potential impact of GnRHR agonist activity on drug safety. We developed a prediction model based on compound data for GnRHR agonist activity provided by the Tox21 program and evaluated its performance using nested cross-validation. The model was applied to drugs listed in the FAERS database, and trends in adverse events associated with predicted GnRHR agonist activity were then analyzed.

## 2. Results

### 2.1. Model Comparison

The four models, namely, RandomForest, LightGBM, XGBoost, and BalancedRandomForest, were compared using the mean values of evaluation metrics obtained in the outer loop of nested cross-validation. BalancedRandomForest achieved the best performance in terms of ROC-AUC, ACC, MCC, and SP, with an ROC-AUC of 0.810 (Table 1), and was therefore selected for subsequent drug screening. Confidence intervals for ROC-AUC and PR-AUC based on out-of-fold predictions from the five outer folds are provided in Supplementary Table S1. A sensitivity analysis excluding PubChem inconclusive compounds yielded improved predictive performance across all algorithms; however, the ranking of models based on ROC-AUC remained unchanged, with BalancedRandomForest maintaining the highest performance (Supplementary Table S2). In addition, the final model retrained using all data was employed in the analysis. Threshold-performance analysis across multiple uncertainty intervals demonstrated that the 0.3–0.7 range provided a favorable balance between applicability-domain coverage and prediction reliability (Supplementary Table S3). Therefore, compounds within this range were excluded from the applicability domain, while those with scores ≥0.7 were regarded as predicted positives.

**Figure 1:**
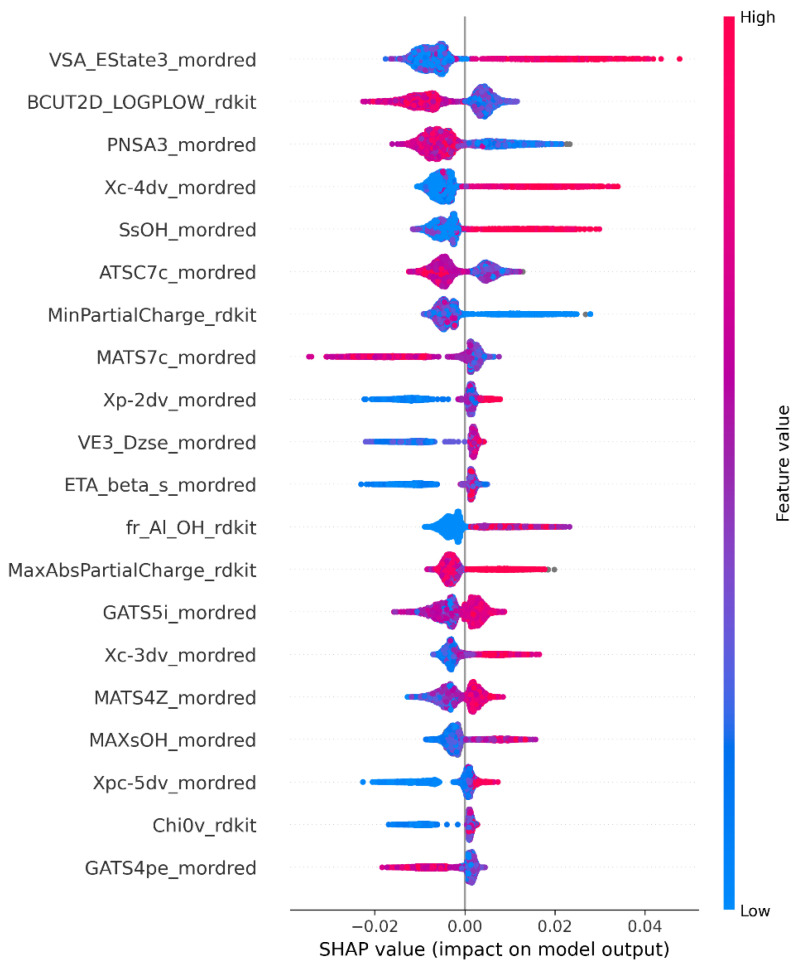
Top 20 features in the GnRHR agonist prediction model obtained by SHAP analysis. Values were calculated according to the contribution of each feature to the model output. SHAP, SHapley Additive exPlanations.

Feature importance was calculated by SHAP analysis using the final model constructed with BalancedRandomForest and all data used for model construction. Descriptors such as fr_Al_OH, SsOH, MAXsOH, PNSA3, VSA_EState3, BCUT2D_LOGLPLOW, GATS5i, GATS4pe, and MATS4Z, which represent solvent interaction properties; MinPartialCharge, MaxAbsPartialCharge, ATSC7c, and MATS7c, which represent local electronic properties; and Xc-3dv, Xc-4dv, Xp-2dv, Xpc-5dv, Chi0v, VE3_Dzse, and ETA_beta_s, which represent skeletal complexity, were the leading features (Figure 1). Higher values of fr_Al_OH, SsOH, MAXsOH, and all skeletal complexity descriptors and lower values of PNSA3, BCUT2D_LOGLPLOW, GATS4pe, ATSC7c, and MATS7c increased the likelihood that a compound would be predicted as a GnRHR agonist.

### 2.2. FAERS-Listed Drug Screening

**Figure 2:**
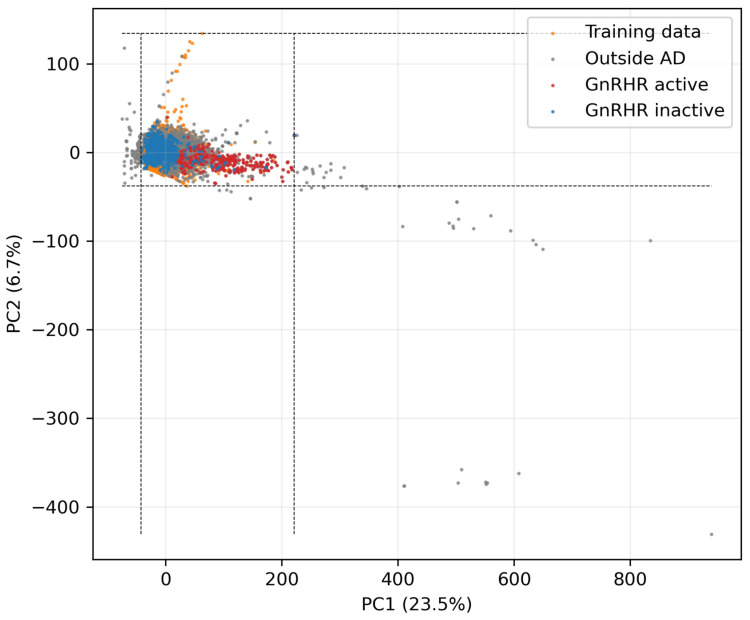
Comparison of the structural distributions of Tox21 compounds and FAERS-listed drugs using principal component analysis. The x-axis represents principal component 1 (PC1), and the y-axis represents principal component 2 (PC2). Orange points indicate Tox21 compounds. Red and blue points indicate FAERS-listed drugs within the applicability domain that were predicted to be active and inactive, respectively. Gray points indicate FAERS-listed drugs outside the applicability domain. The black dashed box, which was used as an indicator of the applicability domain, indicates the range of principal component scores for compounds in the Tox21 database.

In total, 5523 FAERS-listed drugs for which SMILES strings could be obtained served as candidates for GnRHR agonist prediction. When the applicability domain was defined using the minimum and maximum scores of PC1 and PC2 from PCA based on the Tox21 data, 5177 of these drugs were considered within the applicability domain (Figure 2). When drugs with an intermediate normalized score for the active class (0.3–0.7) from the final BalancedRandomForest model were considered outside the applicability domain, 1191 drugs remained within the applicability domain, of which 367 were predicted to be positive for GnRHR agonist activity (Supplementary Table S4). As a supplementary validation of the applicability domain, a distance-based k-nearest neighbors (kNN) approach was also evaluated. Using the kNN criterion, 4093 FAERS-listed drugs were classified as being within the applicability domain. Among these compounds, 157 were predicted to have GnRHR agonist activity (Supplementary Table S4). Although the kNN-based approach yielded a more restrictive applicability domain than the PCA- and score-based approach, a substantial number of compounds predicted to have GnRHR agonist activity remained within the applicability domain, supporting the robustness of the overall findings.

**Figure 3:**
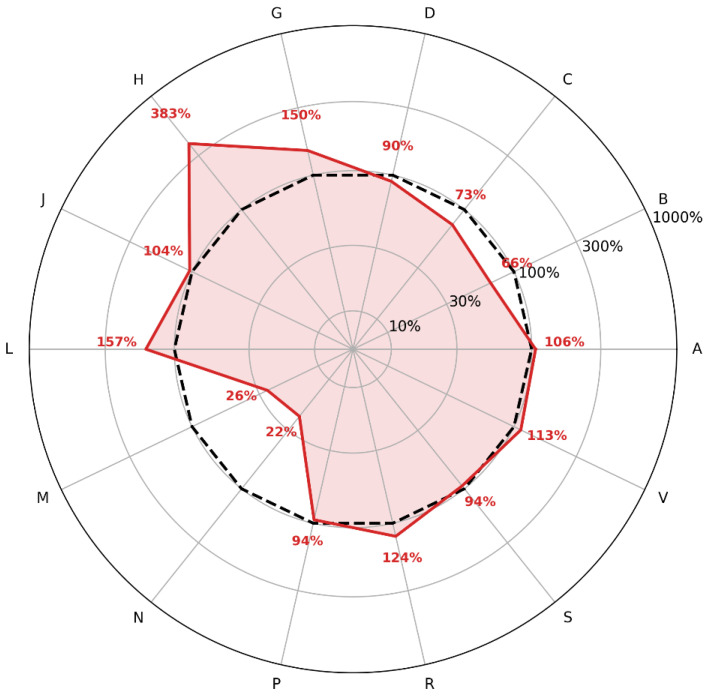
Distribution of drugs predicted to be positive for GnRHR agonist activity by ATC first-level category relative to all ATC-classified, FAERS-listed drugs. The black dashed line represents the 8066 ATC-classified, FAERS-listed drugs. The red solid line represents 193 ATC-classified drugs predicted to be active for GnRHR agonist activity. Letters correspond to ATC first-level categories: A, alimentary tract and metabolism; B, blood and blood-forming organs; C, cardiovascular system; D, dermatologicals; G, genitourinary system and sex hormones; H, systemic hormonal preparations excluding sex hormones and insulins; J, anti-infectives for systemic use; L, antineoplastic and immunomodulating agents; M, musculoskeletal system; N, nervous system; P, antiparasitic products, insecticides, and repellents; R, respiratory system; S, sensory organs; V, various.

To characterize the 367 drugs predicted to have GnRHR agonist activity, we classified the drugs according to the first level of the Anatomical Therapeutic Chemical (ATC) classification system. Among FAERS-listed drugs, 8066 drugs were assigned ATC codes, and 193 drugs were both predicted to exhibit GnRHR agonist activity and assigned ATC codes. We compared the distribution of predicted GnRHR agonists by ATC first-level category with that of all ATC-classified, FAERS-listed drugs, and the proportion in each ATC category among these FAERS-listed drugs was set to 100% (Figure 3). Drugs predicted to have GnRHR agonist activity were overrepresented among systemic hormonal preparations, excluding sex hormones and insulins (H), whereas drugs acting on the musculoskeletal system (M) and nervous system (N) were underrepresented.

### 2.3. Adverse-Event Signals

First, the FAERS DRUG table (127,228,343 rows) and REAC table (54,645,478 rows) were merged to construct a case-based database. In this database, the number of cases after duplicate removal was used as the analytical unit, and for each adverse event, 2 × 2 contingency tables were generated according to the presence/absence of exposure to drugs predicted to be positive for GnRHR agonist activity and the presence/absence of the adverse event. From 18,328,780 cases, cases involving the 367 drugs predicted to be GnRHR agonists were extracted. A total of 330 unique PTs, corresponding to 531 PT-SOC assignments, met the following criteria: at least 1000 reports, ln(ROR) > 1, and *q* < 0.05 (Supplementary Table S5). When adverse events with at least 1000 reports were summarized by MedDRA System Organ Class (SOC) (Table 2), significant adverse events occurred most frequently in respiratory, thoracic and mediastinal disorders (Supplementary Figure S1), infections and infestations (Supplementary Figure S2), and hepatobiliary disorders (Supplementary Figure S3).

## 3. Discussion

### 3.1. Applicability of the GnRHR Agonist Prediction Model

Using GnRHR agonist activity data from the Tox21 database, we developed a machine learning model based on 1761 molecular descriptors. In nested cross-validation, the model achieved a mean ROC-AUC of 0.810, indicating a moderate ability to rank active compounds above inactive compounds. However, the relatively low PR-AUC and MCC indicated that the model’s ability to make definitive positive predictions for individual compounds was limited, particularly given the substantial class imbalance. Accordingly, the model was used to prioritize candidate drugs and generate hypotheses through subsequent FAERS adverse event analysis, rather than to confirm the GnRHR agonist activity of individual drugs. The prediction model’s applicability domain was evaluated from two perspectives: (i) distribution in the chemical space based on PCA and (ii) distribution of normalized model scores. In the PCA-based assessment, the chemical applicability domain was defined according to the range of Tox21 compounds in the PC1–PC2 plane. Many compounds predicted to have high GnRHR agonist activity were distributed within or near this region. This result supports the model’s external validity because predictions were made consistently within the chemical space represented by the training data [18]. However, the variance explained by PC1 and PC2 in this dataset was relatively low (23.5% and 6.7%, respectively); thus, PCA alone may be insufficient to strictly define the applicability domain. Therefore, the distribution of normalized scores was used as a complementary criterion, and compounds with normalized active-class scores within the intermediate range of 0.3–0.7 were considered outside the applicability domain because of high model uncertainty. In classification models, score-based applicability domain definitions reportedly improve predictive performance more effectively than definitions based solely on distance information, including PCA [19]. Thus, the criteria used in the present study align with previous findings. By defining the applicability domain using both chemical space and normalized model scores, the present model enabled us to select more reliable candidate compounds while reducing excessive extrapolation.

In the prediction model, higher values of descriptors related to the number of hydroxyl groups, including fr_Al_OH, SsOH, and MAXsOH, as well as descriptors reflecting skeletal complexity, increased the likelihood of being predicted as a GnRHR agonist. Conversely, lower values of PNSA3 (representing a polar and negatively contributing molecular surface area), BCUT2D_LOGLPLOW (reflecting lipophilicity), GATS4pe (reflecting pi-electronic properties), ATSC7c (reflecting the overall spread of charge), and MATS7c (reflecting charge regularity) were associated with a higher probability of being predicted as a GnRHR agonist. GnRHR contains many polar amino acids and depends on hydrogen bonding and electrostatic interactions [20]. It is also regarded as a distinctive G protein-coupled receptor that selectively recognizes a U-shaped folded GnRH structure in the ligand-binding domain instead of a disordered peptide conformation [21,22]. Furthermore, GnRH acts as a hydrophilic peptide enriched with polar amino acids, with many GnRH analogs containing multiple hydroxyl groups and forming sterically constrained structures through D-amino acid introduction or cyclization. These observations are broadly compatible with the molecular characteristics of GnRHR agonists identified in our study and suggest that the prediction model captures, at least in part, the molecular properties of GnRHR agonists.

Drugs predicted to be GnRHR agonists were enriched among systemic hormonal preparations excluding sex hormones and insulins (H), consistent with the representative GnRHR agonists gonadorelin and nafarelin classified as GnRHs (H01CA). Similarly, antineoplastic and immunomodulating agents (L)—the next most frequent category—include buserelin, leuprorelin, goserelin, triptorelin, and histrelin, which are classified as GnRH analogs (L02AE). In vitro GnRHR agonist activity has also been confirmed in the Tox21 database for adrenergic drugs (e.g., racepinephrine and epinephrine), cholinergic drugs (e.g., acetylcholine), and bile acids (e.g., chenodeoxycholic acid). These compounds were predicted by the present model to have GnRHR activity, given that their normalized scores were above 0.95. Therefore, through this analytical framework, GnRHR-associated adverse-event risks may be evaluable not only for established GnRHR agonists but also for drugs with potential GnRHR agonist activity.

### 3.2. Adverse Events Related to Respiratory, Thoracic, and Mediastinal Disorders

Drugs predicted to be GnRHR agonists were frequently associated with respiratory adverse events in this study. This finding aligns with previous reports and existing evidence linking androgen deficiency to effects on lung tissue.

Several cohort and database studies have reported that GnRHR agonist therapy is associated with increased risks of interstitial lung disease [11], community-acquired pneumonia [23], and radiation pneumonitis [24].

Ojeda et al. assessed morphological and biochemical changes in the lungs of male rats receiving surgical or pharmacological castration. They found that androgen deficiency induced alveolar septal destruction, emphysema-like changes, and alterations in the phospholipid composition of pulmonary surfactant [25]. Rats treated with antiandrogen flutamide also demonstrated similar morphological lung changes. Therefore, androgens are potentially essential for maintaining normal lung morphology and pulmonary surfactant metabolism.

Taken together, androgen deficiency induced by GnRHR agonists may compromise the structural integrity of lung tissue through morphological and biochemical changes in pulmonary cell membranes. This may increase susceptibility to respiratory diseases, including pneumonia.

### 3.3. Adverse Events Related to Infections and Infestations

The high number of reported infection-related adverse events aligns with a series of evidence linking androgens to immune function.

Androgen receptors are expressed in many immune cells, including neutrophils [26], and androgens promote granulocyte progenitor proliferation and increase neutrophil production [27]. Using androgen receptor–knockout mice, Chuang et al. showed that androgen receptor deficiency was associated with neutropenia and increased vulnerability to microbial infection. They also showed that exogenous androgen administration restored neutrophil counts in castrated mice [28].

According to Ojeda et al., androgen deficiency induces changes in the phospholipid composition of pulmonary surfactant and morphological changes in lung tissue [25], potentially impairing airway defense mechanisms. Hicks et al. also showed that androgen deprivation therapy (ADT) significantly increased the risk of hospitalization for community-acquired pneumonia [23]. Thus, the synergistic effects of immune dysfunction and pulmonary tissue changes may increase infection risk among GnRHR agonist users.

### 3.4. Adverse Events Related to Hepatobiliary Disorders

Hepatobiliary adverse events were also frequently reported for drugs predicted to be GnRHR agonists in this study, consistent with the known metabolic adverse effects of ADT.

In a large population-based study, Gild et al. showed that ADT, including GnRHR agonists, was associated with liver disease diagnoses such as cirrhosis, hepatic necrosis, and nonalcoholic fatty liver disease in men with prostate cancer [12]. Saylor et al. also reported that GnRHR agonist therapy significantly increased biliary disease incidence and that the risk increased with longer duration of use, suggesting a dose–response relationship [29]. In addition, bilateral orchiectomy was not associated with a significant increase in biliary disease risk, suggesting that mechanisms specific to GnRHR agonists contribute to biliary disease.

Moreover, GnRHR agonists induce metabolic abnormalities, including increased fat mass, particularly abdominal fat accumulation, elevated serum triglycerides, and increased insulin resistance [30]. According to a metabolomic analysis, most bile acids and their metabolites increase within 3 months after the initiation of GnRHR agonist therapy [31]. Additionally, hormonal changes can induce gallbladder dysmotility [32]. These factors may have contributed to the increased risk of developing hepatobiliary diseases observed in the present study.

### 3.5. Adverse Events Related to the Reproductive System and Breast Disorders

In the present study, the proportion of reported adverse events classified as reproductive system and breast disorders was relatively low among cases with GnRHR-active drug exposure (Supplementary Figure S4). However, this result may reflect changes in the composition of adverse-event reports related to underlying indications rather than the drugs’ safety profile. Given that GnRHR agonists are mainly used to treat breast cancer [8] and prostate cancer [7], reports related to these diseases may have influenced the overall reporting structure, making the proportion of reproductive system and breast disorders appear relatively low.

Conversely, ovarian hyperstimulation syndrome (ROR = 13.48, *q* < 3.41 × 10^−322^) and polycystic ovaries (ROR = 3.17, *q* = 4.06 × 10^−78^), which are known adverse events linked to GnRHR agonists, were detected as significant signals. These findings align with previous reports on ovarian stimulation and effects on reproductive endocrine function induced by GnRHR agonists [1,2,3,6], supporting the validity of the present analysis.

Therefore, the lower proportion of reported reproductive system and breast disorders may reflect the influence of underlying indications and changes in the reporting structure rather than a reduced risk of reproductive system-related adverse events.

### 3.6. Limitations

This study has several limitations that need to be considered when interpreting and generalizing the findings. First, machine learning models based on QSAR may be biased by the training data’s structural distribution, and their predictive performance for structurally distinct unknown compounds may be limited. Furthermore, some molecular descriptors are expected to be highly correlated, potentially affecting the model’s interpretability and reproducibility [33]. In addition, the 3D molecular descriptors were calculated from MMFF-minimized conformers, which may not represent the actual receptor-bound conformations of ligands.

Second, given that Tox21 activity data are based on in vitro assays, the direct relationship of these data with in vivo pharmacological activity or actual adverse effects remains unclear. In addition, the inclusion of PubChem inconclusive compounds in the negative class may have introduced label ambiguity and reduced class separability, which could have affected the model’s generalizability and classification performance. These limitations affect how the prediction results are interpreted clinically. Third, the model was applied to 1191 drugs for which SMILES strings were available and were within the applicability domain of the Tox21-based prediction model. Consequently, other drugs were excluded from the prediction results, and the influence of these excluded compounds on the overall evaluation remains unknown.

Fourth, the present analysis was conducted at the MedDRA SOC level and may not fully capture variation among individual adverse events. Pharmacovigilance research may exhibit a masking effect, wherein a large number of other adverse-event reports obscure specific adverse-event signals [34,35]. Indeed, in our analysis, the proportion of reported reproductive system and breast disorders was low at the SOC level, while significant signals were detected for individual PTs, including ovarian hyperstimulation syndrome and polycystic ovaries. Therefore, SOC-level aggregation may not fully capture detailed changes in adverse-event profiles.

Fifth, spontaneous reporting databases record only cases with reported adverse events and without information on all patients exposed to a drug [36]. Thus, the incidence of adverse events cannot be calculated directly, and indicators such as ROR must be used for risk evaluation, thereby potentially introducing bias. Sixth, reporting bias must be considered. Some adverse events, particularly mild ones, or newly emerging risks may be underreported, whereas serious adverse events or known risks may be overreported or underreported, likely affecting the results [36,37]. Seventh, indication bias and channeling bias should be considered when interpreting the results. The observed disproportionality signals may be influenced by the underlying diseases, patient characteristics, and clinical contexts for which the drugs were prescribed [34]. Furthermore, patients may be systematically selected for GnRHR agonist treatment on the basis of clinical characteristics, including disease severity, comorbidities, concomitant therapies, and monitoring intensity, potentially affecting the observed reporting patterns.

Finally, when multiple drugs are used concomitantly, identifying which drug caused an adverse event is difficult [36,38]. Our analysis included drugs recorded as PS, secondary suspect, concomitant, or interacting, thereby potentially leading to increased FP findings. Therefore, causal relationships between drugs and adverse events cannot be established from this study alone. Further research that accounts for these factors is warranted.

## 4. Materials and Methods

### 4.1. Data Source

We collected data on GnRHR agonists from the Tox21 database (AID: 1920067; https://pubchem.ncbi.nlm.nih.gov/bioassay/1920067; accessed on 6 January 2025) to comprehensively analyze compounds with GnRHR agonist activity. Based on high-throughput screening, Tox21 is a large-scale toxicity database developed for an efficient and predictive assessment of chemical toxicity [14]. From this database, compounds with Simplified Molecular Input Line Entry System (SMILES) strings and activity scores were included, whereas those with duplicate SMILES strings and without carbon atoms were excluded. Subsequently, 7133 compounds were included in the analysis. For compounds with duplicate SMILES strings, the median PubChemScore was calculated. According to PubChem criteria, compounds with PubChemScore ≥40 were considered positive (label: 1), whereas those with PubChemScore <40 were considered negative (label: 0). The negative class included 252 compounds designated as inconclusive by PubChem (activity score ≥1 and <40). Ultimately, the final dataset comprised 7071 negative and 62 positive compounds.

### 4.2. Molecular Structures and Chemical Descriptors

RDKit, an open-source cheminformatics toolkit, was used for cleaning and standardizing SMILES strings [39] by removing salts, counterions, and fragments and adjusting protonation states by neutralization. Considering the computational burden associated with a multitude of candidate compounds, optimal three-dimensional (3D) structures were generated using an efficiency-oriented heuristic procedure. First, chemical structures were generated from the SMILES strings, and explicit hydrogen atoms were added. Next, up to 200 3D conformers were randomly generated. Each conformer was energy-minimized using the Merck molecular force field (MMFF), and the one with the lowest energy was selected. If this process exceeded 60 s, conformers were generated using the Experimental-Torsion Knowledge Distance Geometry method [40], followed by energy minimization with MMFF [41]. The selected conformer was then converted into a structure-data file (SDF) format.

For each compound, molecular descriptors were calculated using the Python libraries RDKit and mordred-community, an actively maintained fork of Mordred [42,43]. Two-dimensional and 3D descriptors, including RDKit descriptors (221 dimensions) and Mordred descriptors (1826 dimensions), were obtained. From a total of 2047 descriptors, perfectly correlated descriptors and those with zero variance were removed, leaving 1761 descriptors for model construction. These preprocessed descriptors were used for machine learning model development without further scaling. For PCA, the descriptor values were standardized to zero mean and unit variance prior to analysis.

### 4.3. Model Development

Classification models were developed to predict GnRHR agonist activity. Four algorithms were compared: RandomForest [44], LightGBM [45], XGBoost [46], and BalancedRandomForest [47]. The 1761 molecular descriptors calculated using RDKit and Mordred served as the explanatory variables, whereas the presence/absence of GnRHR agonist activity, as defined by PubChemScore, was the objective variable.

As an external-validation-like framework, model performance was evaluated using nested cross-validation to estimate generalization performance while reducing overfitting [17]. Specifically, stratified *k*-fold cross-validation was performed using five outer and four inner folds. We used the outer folds for evaluating generalization performance and the inner folds for optimizing hyperparameters. Hyperparameters were optimized using Bayesian optimization with Optuna [48]. The optimization process included class imbalance handling parameters, where the sample weight assigned to positive samples was varied from 1 to 10 times that assigned to negative samples for all algorithms. For BalancedRandomForest, the sampling_strategy parameter was additionally optimized. For each algorithm, 100 Optuna trials were conducted, and the parameter set with the highest area under the receiver operating characteristic curve (ROC-AUC) was selected. In comparing models, the algorithm with the highest mean ROC-AUC across the five outer folds was selected for the final model. The final model was retrained using all data. A total of 100 hyperparameter patterns were explored with Optuna; the parameter set that achieved the highest ROC-AUC in fivefold cross-validation was selected as the final setting. Feature importance was then calculated by SHapley Additive exPlanations (SHAP) analysis using the final model and all data used for model construction [49].

### 4.4. Evaluation Metrics

The classification models’ predictive performance was evaluated according to the number of true positives (TP; positive compounds correctly predicted as positive), true negatives (TN; negative compounds correctly predicted as negative), false negatives (FN; positive compounds misclassified as negative), and false positives (FP; negative compounds misclassified as positive). The following seven metrics were used for the evaluation.

Sensitivity (SE): SE is the proportion of actual positive samples correctly predicted as positive.

(1) $$SE=\frac{TP}{TP+FN}$$

Specificity (SP): SP is the proportion of actual negative samples correctly predicted as negative.

(2) $$SP=\frac{TN}{TN+FP}$$

Accuracy (ACC): ACC is the number of correctly predicted samples divided by the total number of samples.

(3) $$ACC=\frac{TP+TN}{TP+TN+FP+FN}$$

Balanced accuracy (BA): BA is the mean of SE and SP.

(4) $$BA=\frac{1}{2}\left(SE+SP\right)$$

Matthews correlation coefficient (MCC): MCC is a metric for evaluating classification performance in imbalanced datasets [50].

(5) $$MCC=\frac{TP\times TN-FP\times FN}{\sqrt{\left(TP+FP)(TP+FN)(TN+FP)(TN+FN\right)}}$$

ROC-AUC: ROC-AUC is calculated as the area under the ROC curve based on SE and 1 − SP across thresholds [51].

Average precision (AP): AP is calculated as the area under the precision–recall curve based on precision [TP/(TP + FP)] and recall (SE) across thresholds [52].

The optimal cutoff value for TP, FN, TN, and FP was determined using Youden’s index, which maximizes SE + SP − 1 [53]. Given that the final prediction model had a model-specific cutoff, the model output probabilities were standardized for drug prediction using the following equation:

(6) $$X_{n}=X_{u}^{-\log_{c}2}$$

where *X_n_* represents the normalized score, *X_u_* represents the unnormalized model output probability, and *c* represents the cutoff value determined for the final prediction model. The exponent term (−log_c_2) was chosen so that the model-specific cutoff value was mapped to a normalized score of 0.5. Consequently, prediction scores from models with different cutoff values could be interpreted on a common scale, with values above 0.5 indicating predictions above the model decision threshold and values below 0.5 indicating predictions below the threshold. This normalization facilitated consistent interpretation and comparison of prediction scores for FAERS-listed drugs. It is intended for score rescaling rather than probability calibration.

### 4.5. FAERS Database

**Scheme 1:**
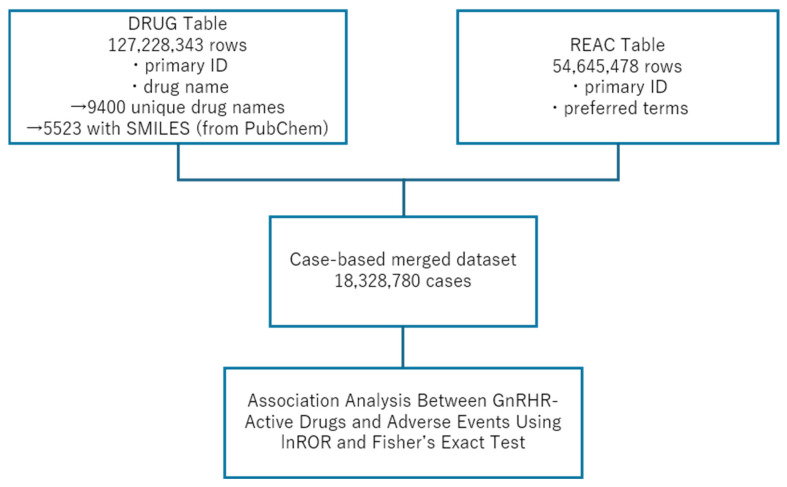
Overview of the FAERS data integration and disproportionality analysis workflow. SMILES, Simplified Molecular Input Line Entry System; ID, identification; GnRHR, gonadotropin-releasing hormone receptor; ln(ROR), natural logarithm of the reporting odds ratio.

The FAERS database was accessed from the official FDA website [54], and data recorded from the first quarter of 2004 through the third quarter of 2024 were used. Adverse events in the reaction (REAC) table are registered according to MedDRA Preferred Terms (PTs), and drug names in the DRUG table were curated by Intage Healthcare Co., Ltd. (Tokyo, Japan). Each case is assigned a PRIMARYID, allowing the integration of data tables. In the analysis, the DRUG and REAC tables were merged by PRIMARYID. When the same drug and the same adverse event were recorded multiple times within the same case, the drug–adverse-event combination was counted only once. This analysis also included drugs recorded with drug roles such as primary suspect (PS), secondary suspect (SS), concomitant (C), or interacting (I) (Scheme 1).

### 4.6. Application of the Prediction Model to FAERS-Listed Drugs

Using the PubChemPy library (https://github.com/mcs07/pubchempy; accessed on 13 June 2025), we searched PubChem to obtain SMILES strings for the 9400 drug names recorded in the FAERS DRUG table [55,56]. The constructed GnRHR agonist prediction model was then applied to 5523 drugs with SMILES strings obtained. To ensure consistency and reproducibility in descriptor calculation for numerous drugs, we limited the analysis to drugs whose SMILES structures could be directly obtained from PubChem. Drugs requiring conversion from other structural formats to SMILES were excluded. Model output probabilities were standardized relative to the cutoff value of the final prediction model using Equation 6. Structural similarity between compounds in the Tox21 database and FAERS-listed drugs predicted to have GnRHR agonist activity was evaluated visually using principal component analysis (PCA). For PCA, the 1761 molecular descriptors employed for prediction model construction were used after preprocessing. Tox21 compounds underwent PCA, and FAERS-listed drugs were projected into the same principal component space using the resulting component loadings. We considered that FAERS-listed drugs located within the range of Tox21 compounds in the PC1–PC2 plot were within the model’s chemical applicability domain (AD). As a supplementary validation of the AD, a distance-based AD assessment was additionally conducted using a k-nearest neighbors (kNN) approach. For each compound, the mean distance to its five nearest neighbors (k = 5) in the training set was calculated using the kneighbors function. The AD threshold was defined as the 95th percentile of the training-set distance distribution, corresponding to retention of 95% of the training compounds. Compounds with mean distances below the threshold were considered inside the AD, whereas those exceeding the threshold were considered outside the AD. Furthermore, the distribution of normalized scores was used as a complementary criterion for AD assessment. Several uncertainty intervals were evaluated (Supplementary Table S4), and compounds with normalized active-class scores between 0.3 and 0.7 were considered outside the AD because predictions near the classification boundary were associated with greater uncertainty. The 0.3–0.7 interval was selected as a practical compromise between retaining compound coverage (86.7%) and excluding uncertain predictions. Although a normalized score of 0.5 corresponds to the model decision threshold, only compounds with normalized scores ≥0.7 were considered high-confidence positive predictions and were included in subsequent adverse event analyses. Additionally, labels were corrected according to experimental activity for 50 drugs with Tox21 GnRHR assay data whose model prediction was inconsistent with the observed experimental activity.

### 4.7. Association Analysis Between Predicted GnRHR Agonist Activity and Adverse Events

We constructed 2 × 2 contingency tables to identify adverse-event signals related to GnRHR agonists (Table 3). The unit of the 2 × 2 table was the number of cases. For each MedDRA PT, cases with at least one drug judged to be positive for GnRHR agonist activity were considered to have GnRHR-active drug exposure, whereas cases involving other drugs were considered to have other drug exposure. Each case was counted only once according to the presence/absence of the adverse event of interest. Specifically, **a** was defined as the number of cases with GnRHR-active drug exposure and the adverse event of interest; **b** as the number of cases with GnRHR-active drug exposure but without the adverse event; **c** as the number of cases with other drug exposure and the adverse event; and **d** as the number of cases with other drug exposure but without the adverse event. When any cell in the 2 × 2 table contains zero, the conventional reporting odds ratio (ROR) cannot be calculated. Additionally, estimates become unstable when cell counts are very small. This bias was corrected using the Haldane–Anscombe correction, which adds 0.5 to all cells, to calculate the adjusted RORs [57]. The *p* values were calculated using Fisher’s exact test. Multiple testing was controlled using the Benjamini–Hochberg procedure. Relative reporting tendencies were assessed by detecting adverse-event signals expressed as RORs. This disproportionality analysis identified frequently reported adverse events [58]. For numerical handling, the minimum *p*-value was set to 5 × 10^−324^ and the minimum ROR to 1 × 10^−308^. Volcano plots were generated, with −log(*q*) on the y-axis and ln(ROR) on the x-axis, for the visual interpretation of adverse-event signals.

### 4.8. Statistical Analysis

Statistical data, including RORs, *p* values, *q* values, and 95% confidence intervals, were analyzed using Python version 3.12.7 (Python Software Foundation, Wilmington, DE, USA). Volcano plots were generated using JMP Student Edition 19.1.2 (SAS Institute Inc., Cary, NC, USA). A random seed of 42 was used for all procedures involving stochastic processes to ensure reproducibility. The major Python packages used in this study included RDKit (version 2026.3.2), mordred-community (version 2.0.7), scikit-learn (version 1.6.1), Optuna (version 4.4.0), LightGBM (version 4.6.0), XGBoost (version 3.2.0), imbalanced-learn (version 0.13.0) and SHAP (version 0.49.1). A *q*-value below 0.05 was considered statistically significant.

## 5. Conclusions

A GnRHR agonist prediction model based on the Tox21 database was constructed in this study, achieving an ROC-AUC of 0.810 in nested cross-validation. This model was applied to FAERS-listed drugs, and associations between adverse events and drugs predicted to be GnRHR agonists were comprehensively analyzed. Results indicated that infections and infestations, respiratory, thoracic, and mediastinal disorders, and hepatobiliary disorders were more likely to be reported than other adverse events, suggesting that GnRHR agonist activity may be associated with these adverse-event reporting profiles. These findings highlight the need for early screening of GnRHR agonist activity in drug development risk assessment and postmarketing safety surveillance and may offer guidance in predicting and preventing adverse effects related to GnRHR agonist activity.

## Figures and Tables

**Table 1:** Model comparison based on nested cross-validation.

Model	ROC-AUC	AP	ACC	BA	MCC	SE	SP
BalancedRandomForest	**0.810**	0.170	**0.801**	0.709	**0.101**	0.615	**0.803**
XGBoost	0.794	**0.203**	0.756	**0.718**	0.099	**0.679**	0.756
LightGBM	0.790	0.198	0.757	0.694	0.088	0.631	0.758
RandomForest	0.771	0.189	0.714	0.679	0.076	0.644	0.715

Bold font indicates the best value for each metric. ROC-AUC, area under the ROC curve; AP, average precision; ACC, accuracy; BA, balanced accuracy; MCC, Matthews correlation coefficient; SE, sensitivity; SP, specificity.

**Table 2:** Distribution of statistically significant adverse-event terms by SOC category.

SOC Category	Significant PT Assignments	Total Terms in SOC	Significant Term Rate (%)
**Respiratory, thoracic, and mediastinal disorders**	**77**	**228**	**33.8**
**Infections and infestations**	**81**	**267**	**30.3**
**Hepatobiliary disorders**	**19**	**82**	**23.2**
Product issues	12	72	16.7
Investigations	40	281	14.2
Immune system disorders	24	173	13.9
Renal and urinary disorders	17	124	13.7
Endocrine disorders	10	73	13.7
Cardiac disorders	17	131	13.0
Injury, poisoning, and procedural complications	49	425	11.5
Blood and lymphatic system disorders	12	111	10.8
Surgical and medical procedures	11	104	10.6
Metabolism and nutrition disorders	14	145	9.7
General disorders and administration site conditions	26	303	8.6
Neoplasms benign, malignant, and unspecified (including cysts and polyps)	15	184	8.2
Congenital, familial, and genetic disorders	2	25	8.0
Vascular disorders	23	294	7.8
Skin and subcutaneous tissue disorders	20	273	7.3
Reproductive system and breast disorders	8	120	6.7
Gastrointestinal disorders	19	342	5.6
Musculoskeletal and connective tissue disorders	12	226	5.3
Pregnancy, puerperium, and perinatal conditions	3	60	5.0
Eye disorders	6	125	4.8
Nervous system disorders	10	368	2.7
Psychiatric disorders	4	230	1.7
**Total**	**531**	**4828**	**11.0**

SOC, System Organ Class. Some preferred terms (PTs) are assigned to multiple SOCs in MedDRA. Therefore, the total number of significant PT-SOC assignments (*n* = 531) exceeds the number of unique significant PTs (*n* = 330). Bold indicates the three SOC categories with the highest significant term rates.

**Table 3:** Case-based 2 × 2 contingency table and formula for calculating the ROR.

	Adverse-Event Present	Adverse-Event Absent
GnRHR-active drug exposure	a	b
Other drug exposure	c	d

a, represents cases with GnRHR-active drug exposure and the adverse event of interest; b, represents cases with GnRHR-active drug exposure without the adverse event; c, represents cases with other drug exposure and the adverse event; and d, represents cases with other drug exposure without the adverse event. ROR, reporting odds ratio [(a/b)/(c/d) = a × d/(b × c)]; GnRHR, gonadotropin-releasing hormone receptor.

## Data Availability

The original contributions presented in this study are included in the article/Supplementary Material. Further inquiries can be directed to the corresponding authors.

## Supplementary Materials

The following supporting information can be downloaded at https://www.mdpi.com/article/10.3390/ijms27156675/s1.

## Abbreviations

The following abbreviations are used in this manuscript:

3D	three-dimensional
ACC	accuracy
AP	average precision
BA	balanced accuracy
FAERS	FDA Adverse-Event Reporting System
FDA	Food and Drug Administration
FN	false negative
FP	false positive
GnRHR	gonadotropin-releasing hormone receptor
MCC	Matthews correlation coefficient
MMFF	Merck molecular force field
QSAR	quantitative structure–activity relationship
ROC-AUC	area under the receiver operating characteristic curve
ROR	reporting odds ratio
SE	sensitivity
SHAP	SHapley Additive exPlanations
SMILES	Simplified Molecular Input Line Entry System
SP	specificity
TN	true negative
Tox21	Toxicology in the 21st Century program
TP	true positive
