# Supplementary material for: Investigation of Adverse Events Associated with Predicted GnRHR Agonists Using the FAERS Database
Source: Int J Mol Sci. 2026 Jul 27;27(15):6675. doi: 10.3390/ijms27156675 (PMC13467096; doi:10.3390/ijms27156675)
Supplement: Supplementary file 1 [file ijms-27-06675-s001.zip › FinalFile_Supplementary_figures.pdf]

Supplementary Figures

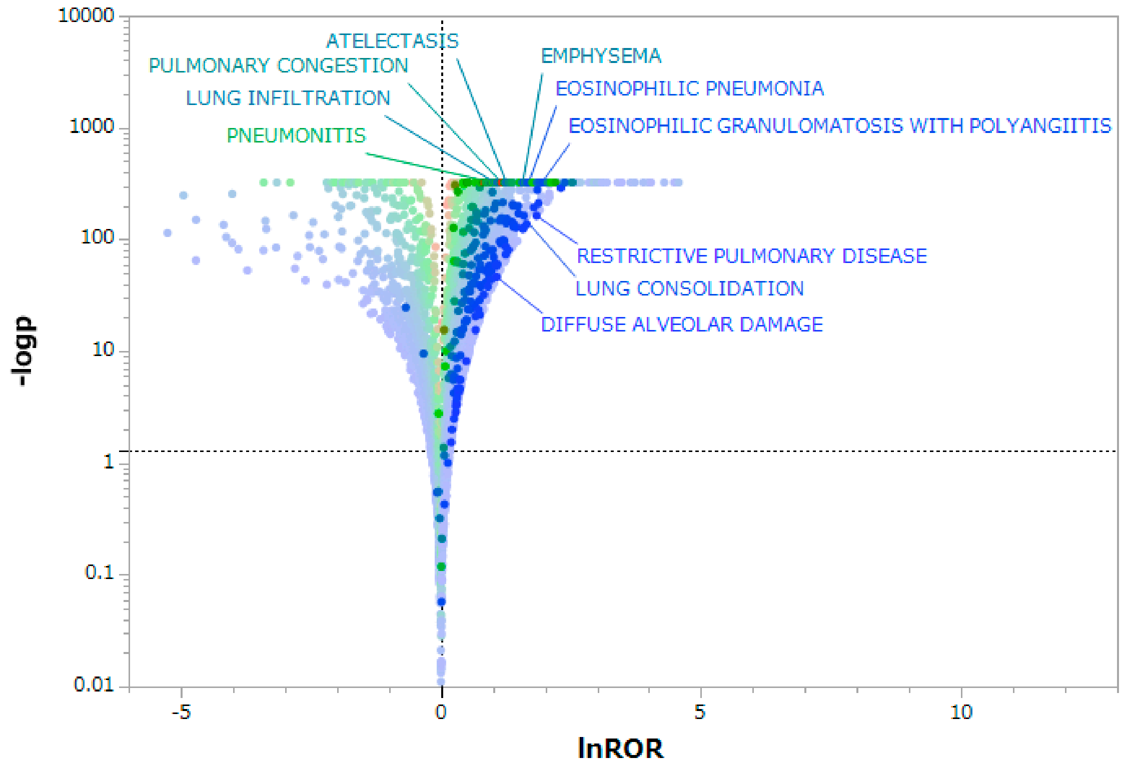

Figure S1. Volcano plot of adverse-event signals in the HLGT category "Lower respiratory tract disorders (excluding obstruction and infection)"

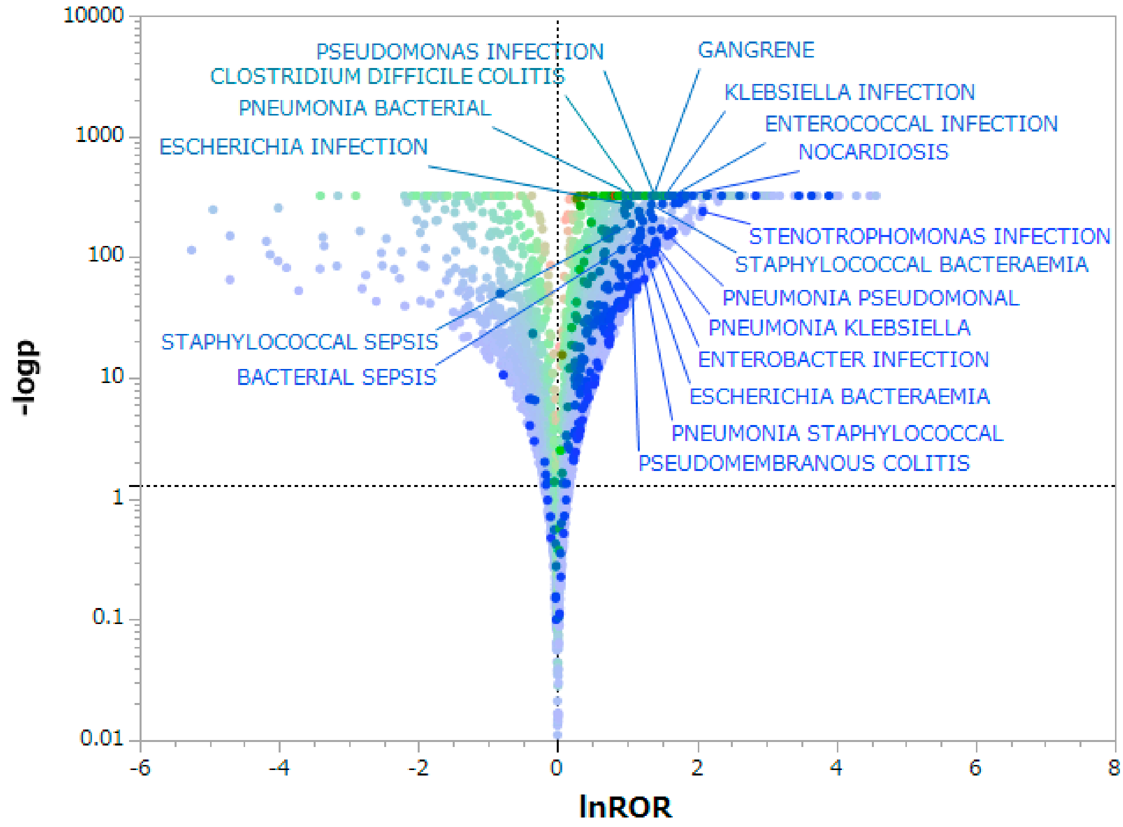

Figure S2. Volcano plot of adverse-event signals in the HLGT category "Bacterial infections."

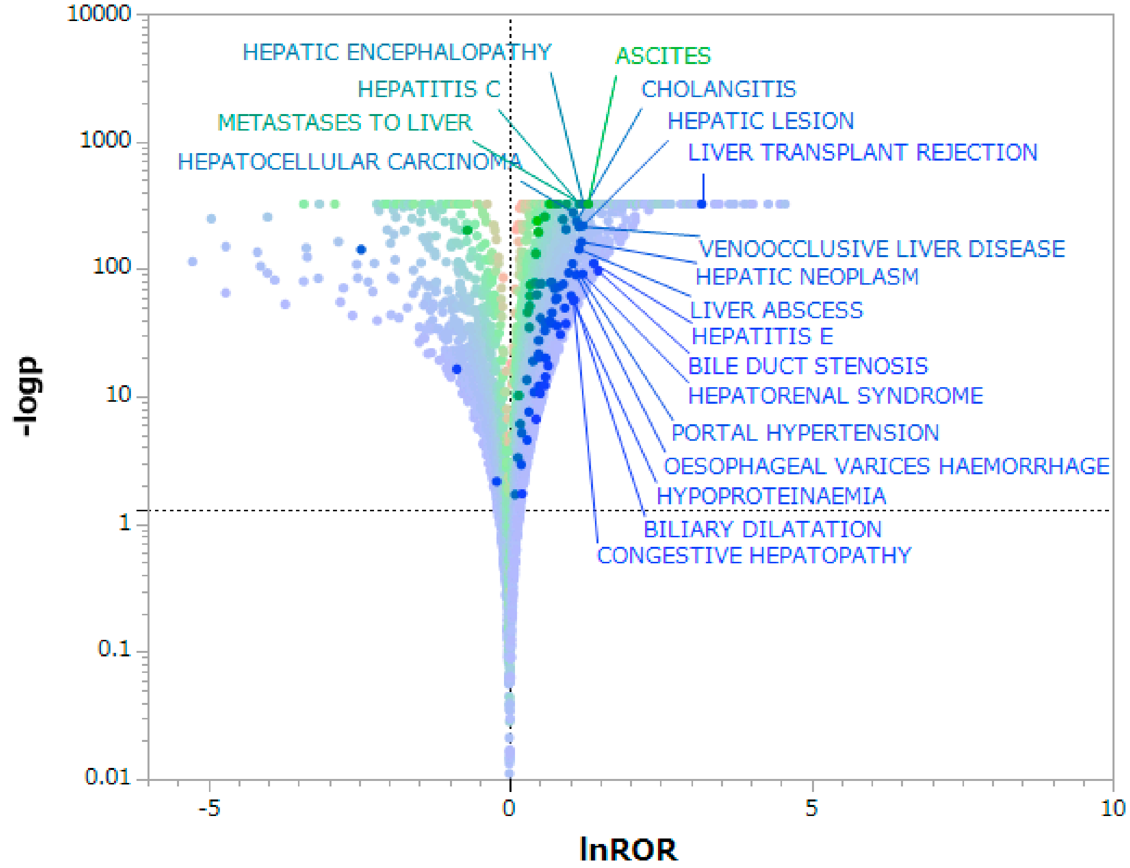

Figure S3. Volcano plot of adverse-event signals related to hepatobiliary disorders.

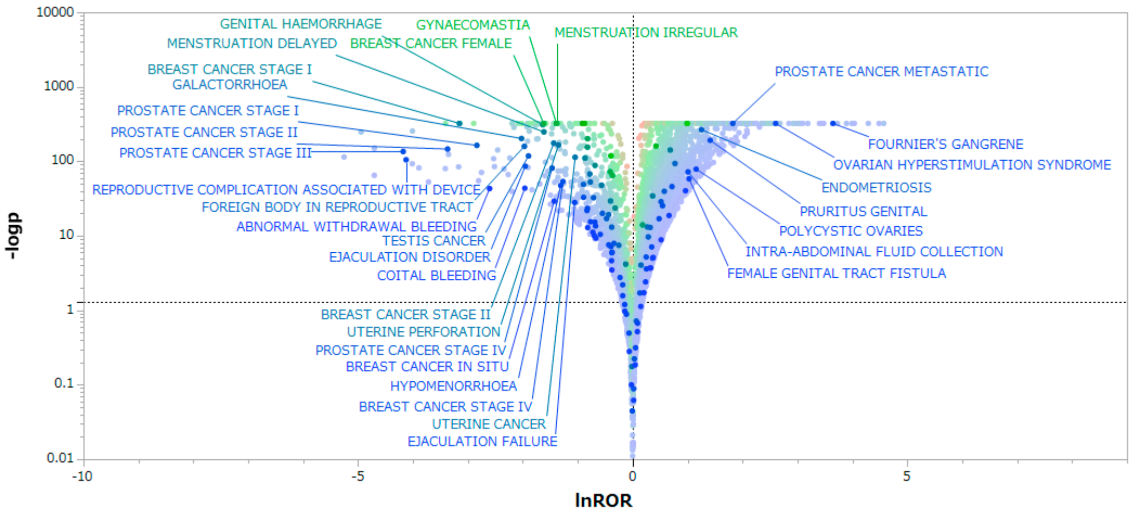

Figure S4. Volcano plot of adverse-event signals related to reproductive system and breast disorders.
